# Diatoms Rely on Sophisticated Signaling Systems for Population Control

**DOI:** 10.1371/journal.pbio.0040089

**Published:** 2006-02-21

**Authors:** Liza Gross

When you're a single-celled organism at the bottom of the food chain, it pays to be resourceful. Diatoms, highly successful photosynthetic plankton responsible for 40% of the net primary production in the oceans, undergo seasonal population explosions called phytoplankton blooms that attract billions of krill, copepods, and other grazing predators. As a defense, wounded diatoms release aldehyde compounds that minimize future diatom casualties by compromising the hatching success of grazers. But these diatom-derived aldehydes can also kill diatoms.

In a new study, Assaf Vardi, Chris Bowler, and their colleagues investigated the possibility that the contrasting effects of aldehydes reflect their role as “infochemicals” that trigger different responses attuned to changing conditions in the diatoms' habitat. The authors found that different concentrations of aldehydes produce different diatom responses. At low doses, aldehydes induce resistance to the compound's toxic effects. High aldehyde concentrations, on the other hand, trigger cell death, which may lead to termination of a bloom. Thus, diatom-derived aldehydes regulate the population dynamics of both diatoms and their predators.

To investigate aldehyde effects on diatom cell fate and population dynamics, the authors studied how two cultured diatom species responded to a highly reactive aldehyde called decadienal. *Thalassiosira weissflogii* is a ubiquitous, cosmopolitan species; *Phaeodactylum tricornutum* is a standard model for understanding diatom biology.

Reactive compounds like decadienal are likely to generate a variety of potentially harmful molecules called reactive oxygen species (ROS). But the authors detected increased levels of just one ROS, nitric oxide, an unstable compound involved in a wide range of physiological processes. Monitoring nitric oxide levels with a nitric oxide–sensitive fluorescent dye and time-lapse imaging revealed that both diatom species experienced similar bursts of nitric oxide production about five minutes after decadienal treatment.

Treated cells succumbed to decadienal in a time- and dose-dependent manner, with significant increases in fatalities above a specific threshold. Below this threshold, cells survived, but underwent cell cycle arrest. To clarify nitric oxide's role in cell death, the authors stimulated nitric oxide production without using decadienal by using molecules called nitric oxide donors. Next, they pretreated cells with a nitric oxide inhibitor before exposing them to decadienal. The number of dying cells increased along with the levels of nitric oxide in the first experiments, and incidence of decadienal-related cell death decreased with the inhibitor. These results clearly implicate nitric oxide in cell death.

How does the cell stimulate nitric oxide production? Since plant and animal cells use calcium to perceive a wide range of environmental signals, Vardi et al. reasoned that diatoms might, too. Using *P. tricornutum* cells that express a calcium-sensitive bioluminescent protein, they tracked changes in intracellular calcium levels in response to aldehydes. As predicted, intracellular calcium levels spiked following decadienal exposure. None of the nitric oxide donors stimulated intracellular calcium production, suggesting that nitric oxide functions downstream of calcium. And, indeed, the nitric oxide synthase that produces nitric oxide was shown to be calcium-activated: after perceiving ambient aldehyde levels, the cell undergoes transient calcium increases that result in nitric oxide production.

Interestingly, nitric oxide production levels varied among the diatoms. Some cells showed rapid increases in nitric oxide production while their neighbors showed delayed responses, suggesting that the signal to produce nitric oxide was propagating through the diatom population. Healthy cells sensed the level of stressed cells in their midst by detecting the wounded cells' aldehyde-generated signal. Cells pretreated with a lower dose of decadienal before receiving a higher dose had far better survival and growth rates than cells treated with only a single high dose. These results suggest that lower decadienal doses may immunize cells, stimulating resistance to normally lethal aldehyde concentrations. This induced resistance may provide diatoms who escape grazing predators with a better chance of surviving the toxic aldehydes released by the dying diatoms.

Altogether, these results suggest that decadienal-like aldehydes not only affect the reproductive capacity of grazers but also act as infochemicals that monitor stress levels in diatom populations. During phytoplankton blooms, this stress surveillance system can induce resistance or death. The authors propose that this differential response, regulated by the sophisticated use of intracellular calcium and nitric oxide signals, may determine the fitness and succession of phytoplankton communities.

The finding that diatoms use chemical signaling for cell–cell communication provides new insights into the cellular mechanisms mediating biotic interactions at the population level and challenges traditional concepts of phytoplankton bloom dynamics. With thousands of different diatom species potentially producing a diverse array of aldehydes, these reactive compounds may prove to be even more versatile than shown here—and may be a key factor contributing to the ecological success of these organisms. Now researchers can begin to unravel the mechanisms that endow one group of molecules with the means to mediate such diverse responses.

## 

**Figure:**
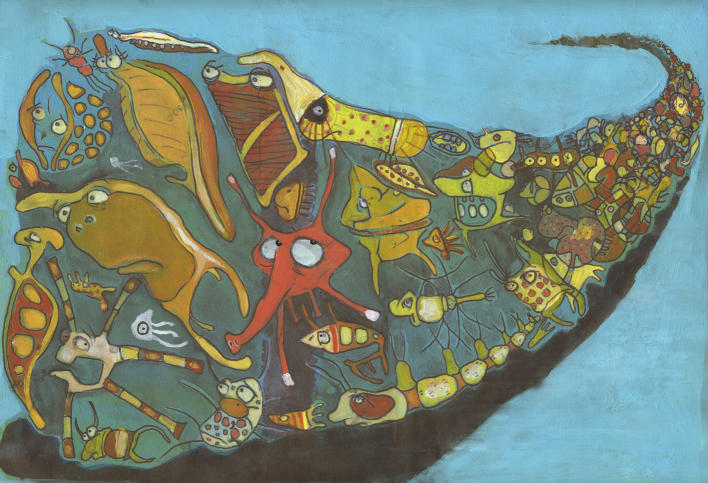
Zooming into the bloom: cell-to-cell signaling in the sea. (Illustration: Nivi Alroy)

